# Engineering Cellular Signal Sensors based on CRISPR-sgRNA Reconstruction Approaches

**DOI:** 10.7150/ijbs.42299

**Published:** 2020-02-21

**Authors:** Hengji Zhan, Lulu Xiao, Aolin Li, Lin Yao, Zhiming Cai, Yuchen Liu

**Affiliations:** 1 Key Laboratory of Medical Reprogramming Technology, Institute of Translational Medicine, Shenzhen Second People's Hospital, The First Affiliated Hospital of Shenzhen University, Shenzhen University School of Medicine, Shenzhen 518035, China; 2 Guangdong Key Laboratory of Systems Biology and Synthetic Biology for Urogenital Tumors, Institute of Translational Medicine, Shenzhen Second People's Hospital, The First Affiliated Hospital of Shenzhen University, Shenzhen University School of Medicine, Shenzhen, 518035, China.; 3 Carson International Cancer Center, Shenzhen University School of Medicine, Shenzhen, 518035, China.; 4 Department of Urology, Peking University First Hospital, Institute of Urology, Peking University, National Urological Cancer Center, Beijing 100034, China.

**Keywords:** CRISPR, signal sensor, riboswitch, sgRNA, synthetic biology

## Abstract

The discovery of the CRISPR systems has enriched the application of gene therapy and biotechnology. As a type of robust and simple toolbox, the CRISPR system has greatly promoted the development of cellular signal sensors at the genomic level. Although CRISPR systems have demonstrated that they can be used in eukaryotic and even mammalian cells after extraction from prokaryotic cells, controlling their gene-editing activity remains a challenge. Here we summarize the advantages and disadvantages of building a CRIRPR-based signal sensor through sgRNA reconstruction, as well as possible ways to reprogram the signal network of cells. We also propose how to further improve the design of the current signal sensors based on sgRNA-riboswitch. We believe that the development of these technologies and the construction of platforms can further promote the development of environment detection, disease diagnosis, and gene therapy by means of synthetic biology.

## Introduction

The cellular signals control the behavior of cells by altering the activity of their genes. The main goal in the field of medical synthetic biology is to create new signaling pathways to allow cells to behave in a pre-determined way. The ability to control cellular behavior has a great impact on human health and medicine. Artificial cellular signal sensors usually include a gene network composed of basic modules that can sense, integrate and process specific biological signals in living cells^1^, ^2^. Although previous studies have demonstrated the advantages of designed signal sensors in many applications including diseases treatment^3^, there are still some problems in the design and assembly of signal sensors, such as the standardization and replacement of required modules, the poor performance of the designed signal sensor, and the interference between the signal sensors and the complex native signaling pathways.

The microbial adaptive immune system clustered regularly interspaced short palindromic repeats (CRISPR) provide a unique and versatile toolbox for targeted genomic engineering^4^-^6^. This robust system is composed of a single CRISPR-associated protein (Cas) and a short fragment of RNA^7^. The most commonly used CRISPR genome editing system is CRISPR-Cas9, which functions with type II nuclease CRISPR-associated protein 9 derived from *Streptococcus pyogenes* (SpCas9). There are various versions that were redesigned to improve one characteristic of this system, such as target specificity^8^, endonuclease activity^9^, or delivery efficiency^10^. Furthermore, other types of Cas9 orthologues, such as Cas12a, Cas12b, Cas13, Cas14 and Cas X, have also been extracted from different bacterial species, and some of which exhibited the advantage of reduced molecular size and improved targeting specificities 11. For example, the recently developed type II CRISPR-associated endonuclease Cas12a, also called Cpf1, has exhibited different characteristics from SpCas9. CRISPR-Cpf1 requires a shorter guiding RNA (gRNA) (only 42-nt) to find its targeted sites instead of the more than 100-nt gRNA of CRISPR-SpCas9. In addition, the T-rich protospacer-adjacent motif (PAM)^12^, ^13^ in the 5' end was recognized by CRISPR-Cpf1 instead of the 3' end of PAM in CRISPR-SpCas9 (TTTV for Cpf1, NGG for SpCas9).

The CRISPR system is composed of a CRISPR- associated endonuclease and related sgRNAs, which can produce gene knock-in or knockout cells from prokaryotic cells to eukaryotic cells. By inactivating the catalytic domain of CRISPR-associated endonucleases, the CRISPR-Cas9/Cpf1 lost the ability to cleave targeted DNAs (dCas9 or dCpf1), and became a transcriptional regulation system for binding the target-specific DNA sequence under the guidance of gRNAs, which expanded the use of the CRISPR system^9^. As with many other DNA-binding proteins, CRISPR systems expand their regulation abilities by fusing different functional proteins with the deactivated CRISPR-associated endonucleases. For example, CRISPR-dCas9/dCpf1 can be engineered to function as transcriptional repressors for CRISPR interference by fusing with transcriptional repression domains, such as the KRAB box^14^, ^15^ or with no further engineering^16^, ^17^. Deactivated CRISPR-associated endonucleases can also serve as active transcription factors^15^, epigenetic modifiers 18, and have been used for DNA/RNA pull-down 19 , high resolution genome imaging^20^ and the real-time detection of pathogenic nucleic acids^3^, ^21^. The regulatory ability and programmability of CRISPR systems provide an useful toolbox for building novel types of cellular signal sensors. Because the CRISPR system contains only two different elements, Cas9 protein and sgRNA, we therefore have two different corresponding strategies for constructing CRISPR signal sensors. Although it is possible to respond to biological signals by engineering Cas9 proteins, this approach is too complex and lacks versatility. Here, we describe the reprogrammed CRISPR signal sensors that were built based on sgRNA-riboswitch reconstruction, which provide an easy implementation and versatile way to sense cellular signals at the genomic level. In theory, they can further regulate any gene of interest within cells flanking a short PAM^22^, ^23^ sequence in response to biological signals.

## Traditional protein based signal sensors

**Figure 1:**
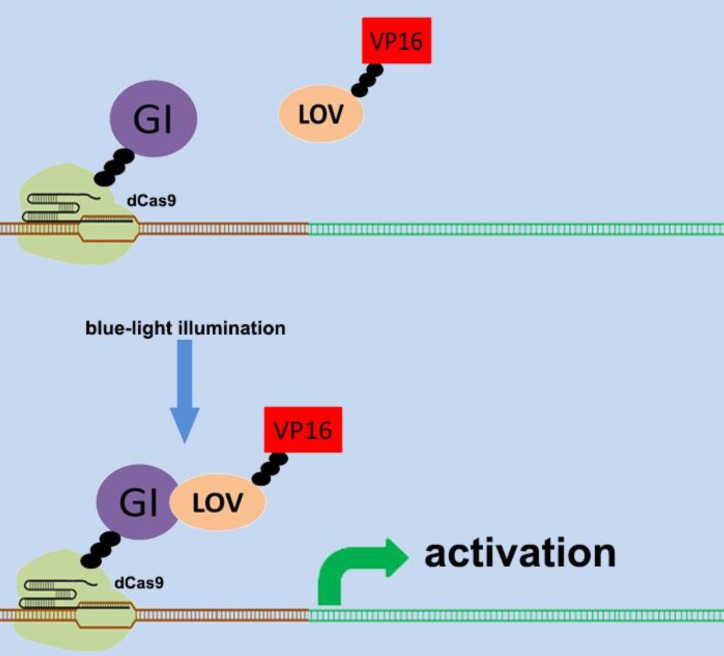
** Design of the CRISPR system for sensing light.** The combination of CRISPR-dCas9 and optogenetics has led to the development of a gene transcriptional regulatory system based on CRISPR-dCas9 that regulates the expression of endogenous genes in response to light. An effective transcription complex can only be formed when exposed to blue light.

There are various types of endogenous signal sensors, which have been previously constructed by synthetic biology and used for the sensing and processing of certain specific endogenous cellular signaling molecules. Specific endogenous signal sensors for certain particular molecules were designed to further construct control systems to interfere with cell behavior. For example, the endogenous signal sensor for the genome guardian P53 protein, which effectively detects the expression level of P53 protein, has been constructed and integrated into gene circuits to screen and eliminate P53-deficient cells^24^, ^25^. Similar sensor-actuator devices were also built based on signal sensors for response to intracellular proteins involved in hepatitis C virus infection, human immunodeficiency virus infection, and Huntington's disease^26^. Another special signal sensor is an optical genetic switch that regulates the expression or activity of the transgene by blue or red light^27^. It should be mentioned that many genetic tools, including CRISPR^28^ and other programmable transcription factors^29^, have also been reengineered for optogenetic regulation (Figure 1). Although these special designs enable efficient response to targeted molecules, these designs can only be used in special situations. It is necessary to establish a universal model for constructing endogenous signal sensors, which work at the genomic level, and can be applied to respond to desired signal molecules. The applications and characteristics of these signal sensors are summarized in Table 1.

## CRISPR-sgRNA based signal sensors

In previous studies, antisense RNAs adapted for aptamer transformation were used as endogenous signal sensors to sense changes in intracellular signals, and these constructs achieved the effect of regulating cell behaviors by regulating target mRNAs 30. This RNA based approach has facilitated the construction of versatile endogenous signal sensors, and certain biological behaviors have been reprogrammed by construction of these simple signal conductors. However, further development of these techniques has been limited due to the instability of RNA in cells and the weak binding ability of antisense RNA to mRNA. It is therefore necessary to construct more stable, effective, and versatile RNA based signal sensors.

**Figure 2:**
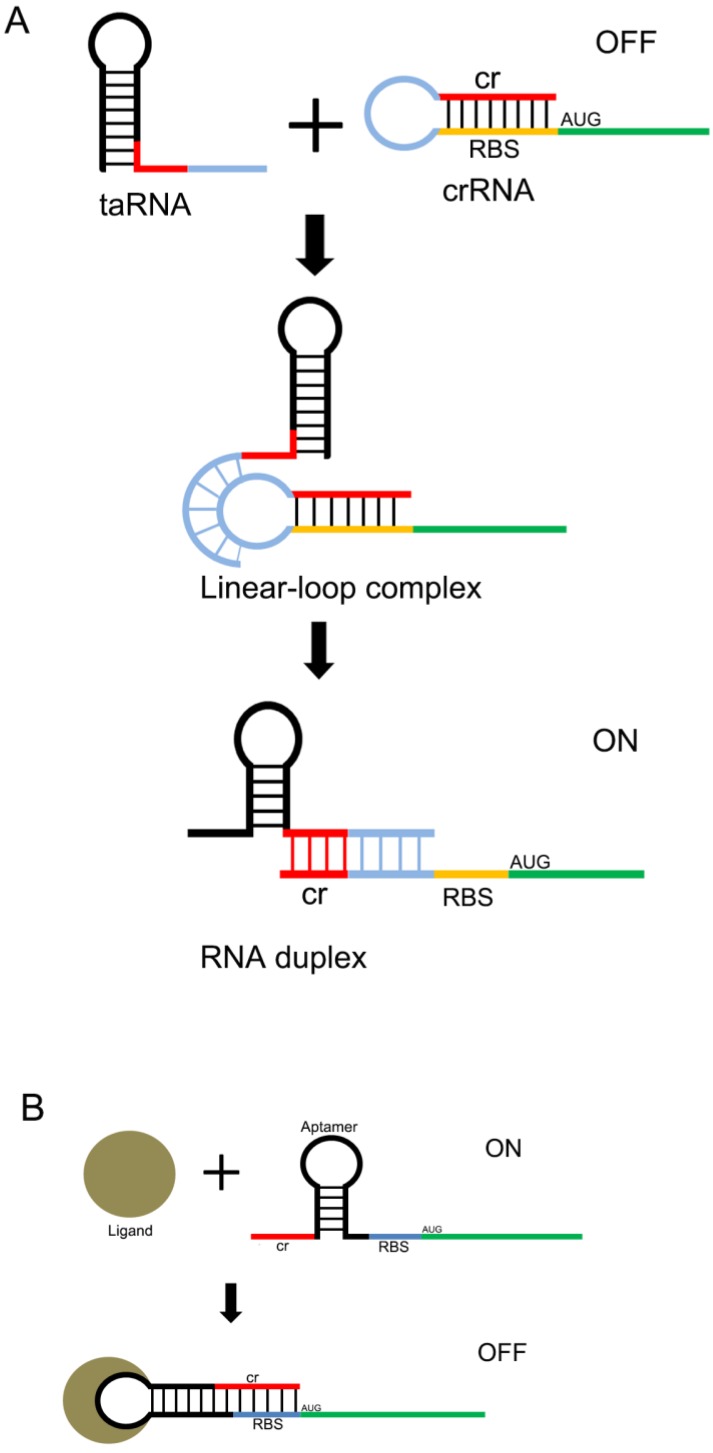
** Design of the riboswitch for sensing molecules.** The ribosome binding site (RBS) of the mRNA 5'UTR is blocked by its own antisense RNA. The RBS site can be exposed only when bound to a specific non-coding RNA(A) or a small molecule(B) , allowing the mRNA to be translated.

Another useful type of RNA signal sensors is the riboswitches discovered in the mRNAs of prokaryotes, which are composed of cis-regulatory modules of RNAs that manipulate the expression of target genes by sensing the specific small signaling molecules within cells. These riboswitches typically contain a domain structure of aptamers, which show high specificity and affinity with the relative ligands (Figure 2 A and B). There are many aptamers that have been discovered in plants, bacteria, and fungi, which show the ability to bind specific molecules selectively and tightly, and regulate the expression of the downstream genes^31^. In natural riboswitches, nucleotide sequences that specifically bind to ligands within the aptamer domain tend to be evolutionarily conserved, and when the ligand is mutated, the nucleotide sequence can also undergo a corresponding change in specificity. These changes are usually achieved by changing the hydrogen bonding patterns responsible for ligand recognition within the aptamer domain. Numerous natural aptamers have been found, which stimulated the design of artificial aptamers. For example, the purine riboswitches^32^, ^33^ have been widely discovered in bacteria, and are found to regulate the expression of orthogonal genes that regulate the cellular behavior and physiological functions of bacteria^34^, ^35^. PreQ_1_, which senses the queuosine precursor, regulates the function of transfer RNA (tRNA) in bacteria^36^, ^37^. The flavin mononucleotide riboswitches, which were extracted from roseoflavin, were found to be related to the antibacterial activity of roseoflavin^38^.

**Figure 3:**
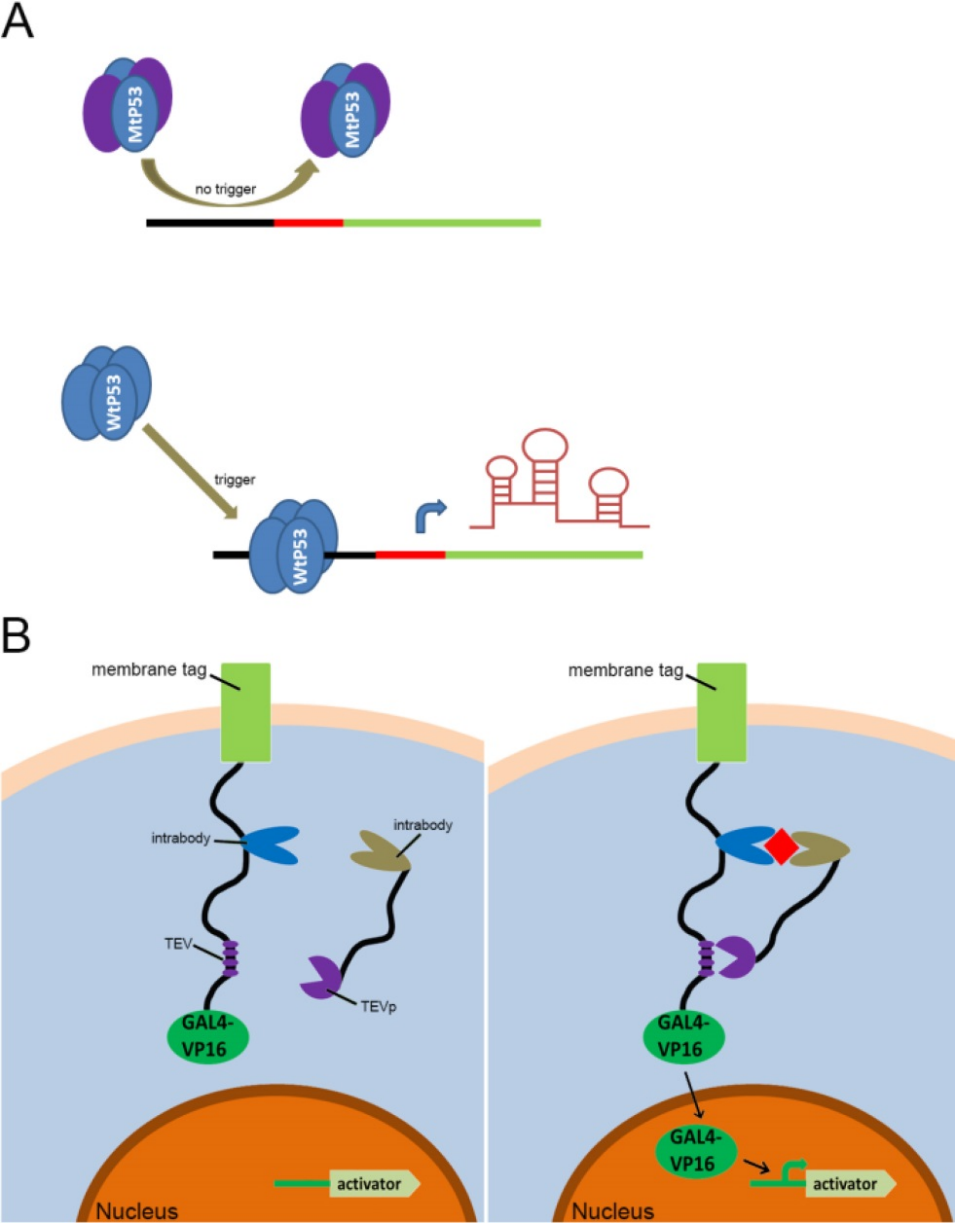
** Previous types of CRISPR signal sensors for sensing specific proteins.** (A) The P53-related signal sensor is unable to recognize mutated P53 proteins (MtP53) in cells, thus rendering the sensor silent in the P53-deficient cells. While the wild type P53 proteins (WtP53) specifically activate the P53-related signal sensor which would turn on the artificial genetic circuit to protect cells from damage. (B) The intracellular protein signal sensor mainly composed of two parts located in the cell membrane and inside the cell. One part is anchored to the membrane and fused at the C-terminus to the TEV cleavage site (TCS) which has already associated with a GAL4-VP16 transcriptional activator. The other part is fused to TEV protease (TEVp). Interactions between the two parts and the target proteins result in the TEVp-mediated release of membrane anchored GAL4-VP16 and output expression.

**Figure 4:**
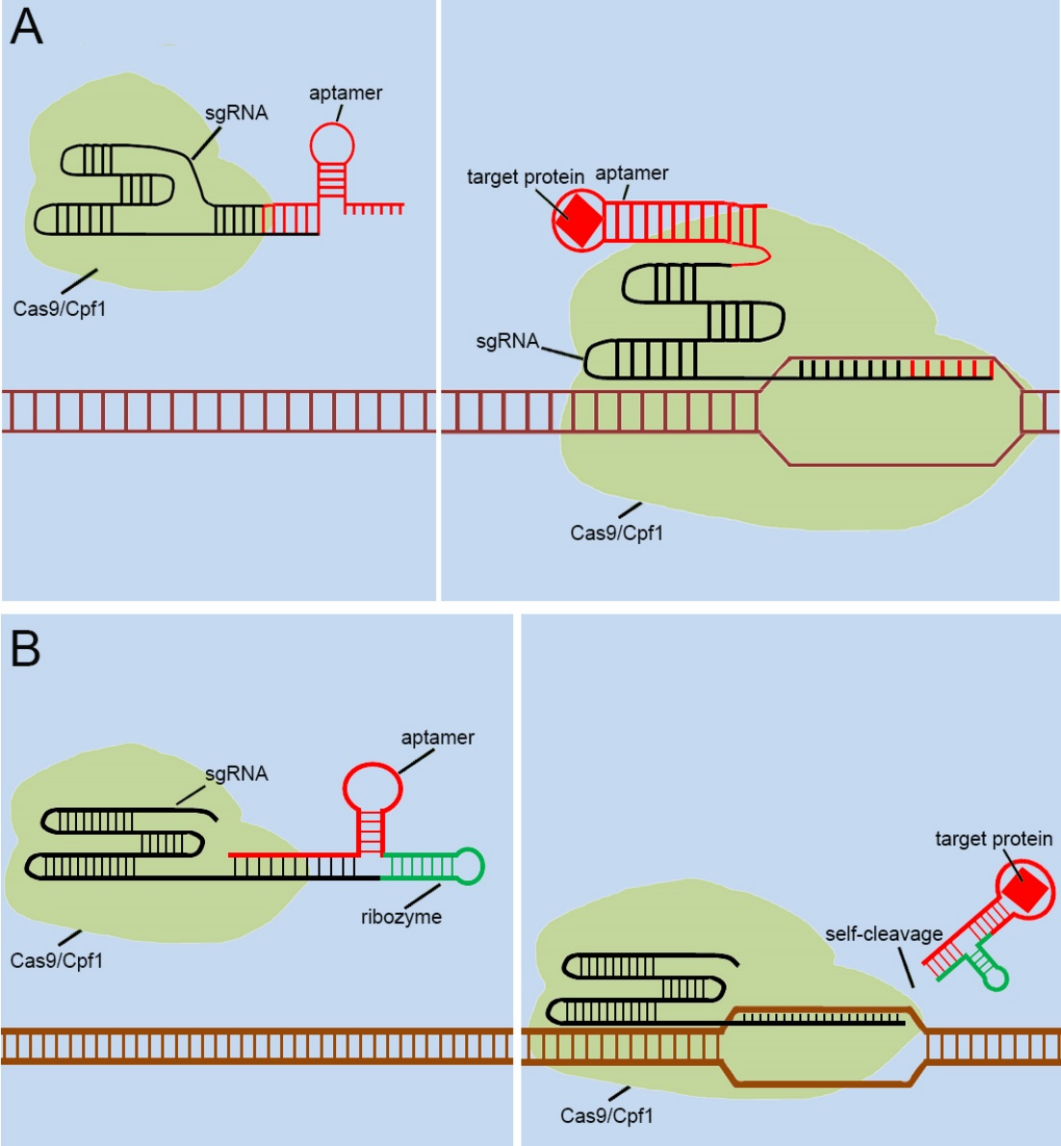
** Previous types of CRISPR signal sensors based on sgRNA-riboswitch.** (A) The additional corresponding sequence which contained aptamer-based riboswitch in the 3'-end of the sgRNA. (B) The additional corresponding sequence which contained aptamer-based ribozyme set in the 5'-end of the sgRNA.

With the discovery and application of the CRISPR system, endogenous signal sensors based on the sgRNA-riboswitch approaches have been gradually developed. We have constructed signal sensors successfully in eukaryotes using CRISPR-Cas9^39^ (Figure 3A) and CRISPR-Cpf1^14^ (Figure 3B), in previous studies in our laboratory. However, these molecular tools either sense only a specific protein or are too complex to make and operate. As mentioned above, riboswitches have been extensively engineered by integrating aptamers that trigger the “on” or “off” signal by a change produced when ligands bind to aptamers. Inspired by riboswitch and antisense RNA regulatory devices, we decided to introduce riboswitch module into sgRNA, which also includes an antisense RNA region at its 5'end. Therefore, CRISPR-sgRNA was engineered using a similar strategy by our group and other teams. The sgRNA that integrates the riboswitch properties allows the entire CRISPR system to acquire the ability to sense related ligands to regulate the “on-off” of the CRISPR system. Since the publication of our studies, there have been many studies attempting to modify sgRNAs in different ways to regulate the CRISPR system 40-42, depending on specific ligands. The interactions of ligands and the aptamers stabilize the structure of the aptamers, and this conformational change exposes the spacer of sgRNA blocked by the aptamer, which allows the sgRNA to restore the ability to guide the Cas protein to the targeted DNA sequence (Figure 4A). Ribozymes and aptamers could be combined to comprise an effective riboswitch, which can serve as the additional corresponding sequence to the 5' end of the sgRNA, to control the “on-off'” of the recombinant sgRNA. When the corresponding ligand is present, this allosteric sgRNA will liberate the additional corresponding sequence at the 5' end and release a working sgRNA because of self-cleavage after the activation of ribozyme, which can convert the closed sgRNA into an open sgRNA 43, 44 (Figure 4B). In theory, any ligand-responsive riboswitch can be used to engineer sgRNA for sensing the specific signal molecules within cells. Many developed aptamers that could be used for constructing ligand-inducible riboswitches have been integrated into endogenous signal sensors based on the CRISPR system.

## New approaches and future designs for sgRNA-riboswitch based signal sensors

**Figure 5:**
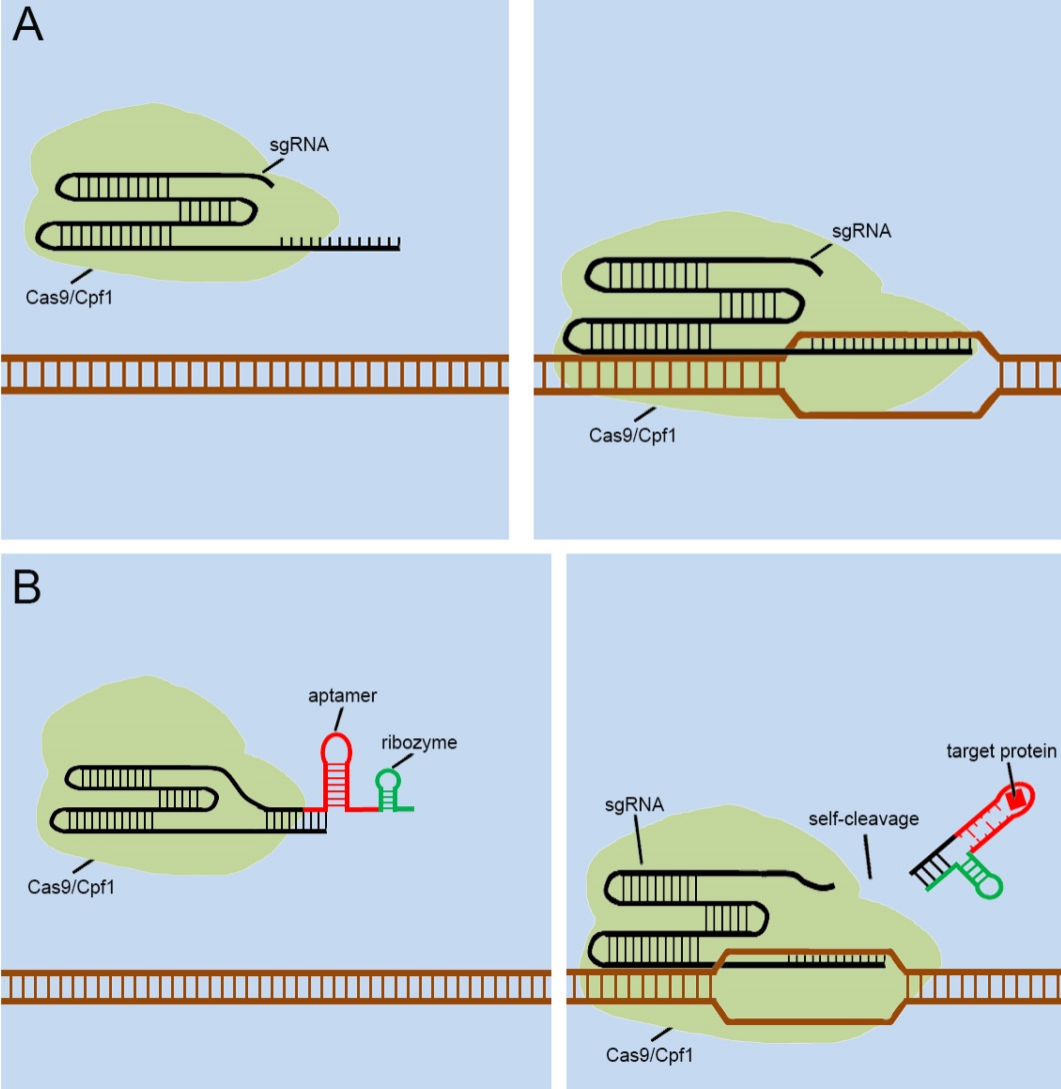
** Future designs of CRISPR signal sensors for detecting proteins of interest.** (A) Original CRISPR-Cas9/Cpf1 versions work directly on DNA, but they cannot be regulated. (B) Ribozymes are added to additional corresponding sequences to participate in the aptamers-mediated regulation of sgRNAs. The interactions between Ligand and aptamer results in a structural change in the additional corresponding sequence, which activates the ribozymes to induce self-cleavage and thus the additional corresponding sequence was automatically released from the sgRNA.

As mentioned above, many groups [40.41,42,43,44] have designed signal transducers for endogenous signals within cells, termed the “CRISPR-endogenous signal sensor,” which sensed the specific signal within the cell and changed the conformation of the sgRNA from the silent state to a functional state (Figure 5A). Interestingly, the designed CRISPR-endogenous signal sensors could sense intracellular protein signals. RNA aptamers that specifically bind to protein signals and form stable structures are an important part of the sensor construction. Previous studies have important implications for further engineering of sgRNAs to sense signaling molecules in cells at the genomic level. The previous modifications to sgRNAs have been largely dependent on aptamer-modified riboswitches. The aptamer-modified riboswitches tend to serve as an additional corresponding sequence, which is attached to the 5' or 3' end of the sgRNA to cover the spacer region of sgRNA, to silence the entire CRISPR system. The additional sequences tend to result in two diverse responses after interacting with the ligands; one involves a complex that remains in the sgRNA, and the other is separated from the sgRNA in some way. The further design of CRISPR-endogenous signal sensors based on the modification of sgRNA should also follow this principle.

However, the previous methods of blocking the sgRNA spacer are not thorough and complete, and may cause Cas9 activity leakage. To resolve this problem, the self-cleavable ribozyme could be integrated into the design of CRISPR-endogenous signal sensor, which allowed the additional corresponding sequence to be automatically cleaved and detached from the sgRNA precursor upon the interaction with the target ligand and aptamers (Figure 5B). In this way, we aimed to optimize our previous design^39^ and reduce the influence of Cas9 activity leakage caused by poor control of sgRNA.

**Figure 6:**
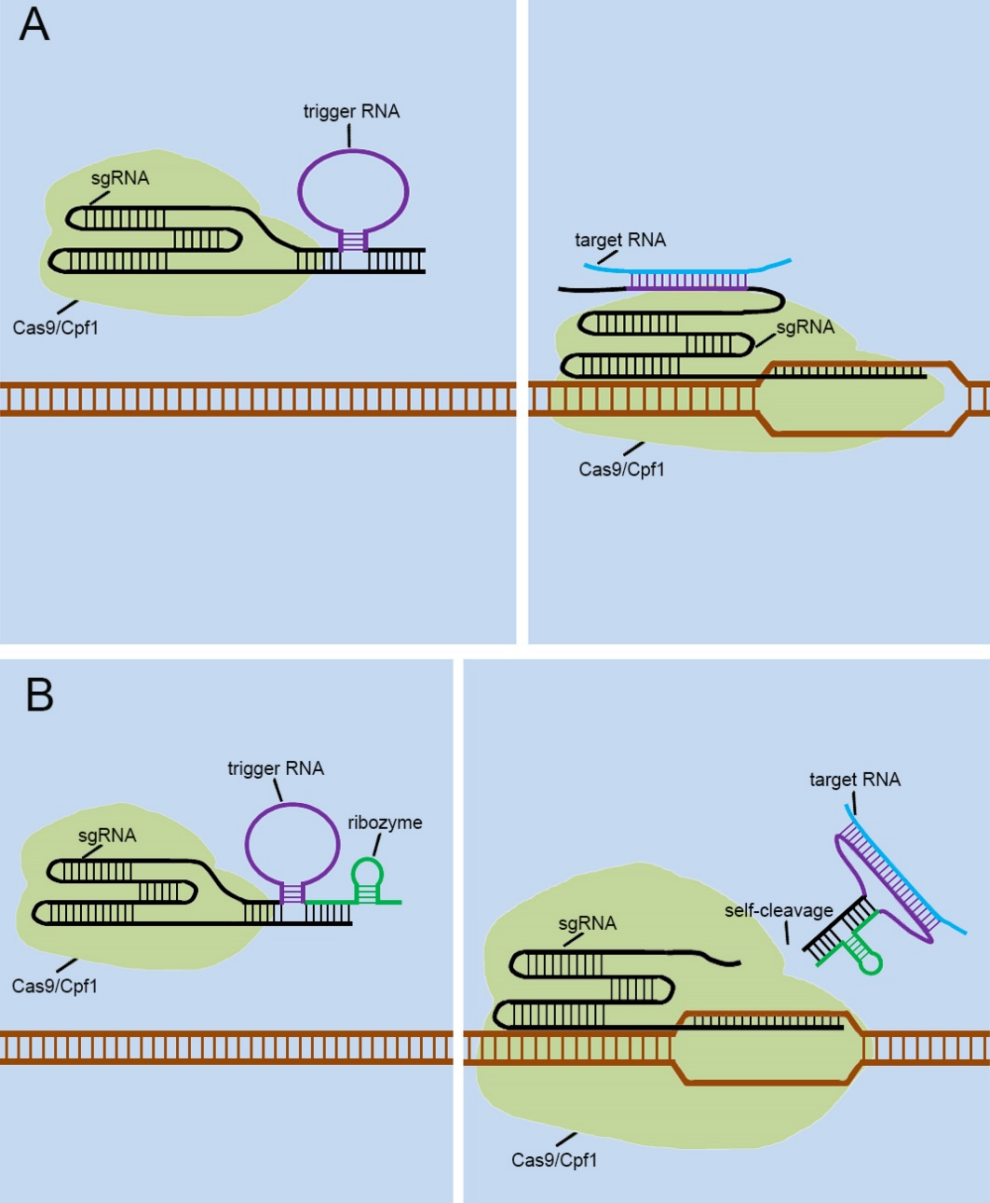
** Future designs of CRISPR signal sensors for detecting RNA molecules of interest.** The detection of RNA molecules is accomplished by the specific trigger RNA, a corresponding antisense RNA complementary to the target RNA molecule. The trigger RNA can be inserted directly into the 3'-end of the sgRNA (A). Also, it can be effectively combined with ribozyme at the 3'-end of sgRNA (B).

Currently, many researchers are also developing some CRISPR-endogenous signal sensors that sensed intracellular RNAs, which regulated the “on-off” sensor in response to the expression levels of specific non-coding RNAs with cells. To achieve this, they designed the riboswitch that responses to RNA transcripts (RNA-switch) based on previously reported RNA reaction devices^45^, and integrated it into sgRNA. The first approach was to attach the RNA-switch to the 3' end of the sgRNA and rely on the RNA-switch to bind to the spacer region of the sgRNA to form the silent state of the RNA-switch-based CRISPR-endogenous signal sensor (Figure 6A). Target RNA transcripts specifically bind to the RNA-switch and alter its conformation, which exposes the spacer region and enables the CRISPR-endogenous signal sensor to successfully perform the corresponding gene editing functions. Another method for constructing CRISPR-endogenous signal sensors that recognize RNA is to use the RNA-switch modulating ribozyme method for the transformation of sgRNA 46. The engineered sgRNAs with regulatory elements composed of the RNA-switch and ribozymes to construct the CRISPR-endogenous signal sensors (Figure 6B). When the target RNA bound to the RNA-switch, the RNA-switch underwent a conformational change to activate the ribozyme, which caused this additional corresponding sequence to be detached from the sgRNA.

The validity of these signal sensors based on sgRNA reconstruction has been tested in both CRISPR-Cas9 and CRISPR-Cpf1 platforms. In addition, our group has recently used these CRISPR- endogenous signal sensors to recode the signaling network within tumor cells to create a novel and effective tumor treatment. Many researchers are currently trying to use similar design strategies to apply our CRISPR signal sensor to metabolic disorders and other diseases. This kind of device can activate different downstream genes by detecting various disease-related markers, so as to correct and make up for the insufficiency of cell function in disease state.

## Conclusions and Outlooks

As mentioned above, the CRISPR system has been engineered for widespread applications, and it has been redesigned recently as the signal sensor for intracellular signal molecules, which can sense small molecules within the cell and trigger the related gene editing activities. Non-coding RNAs have been widely used after being engineered, and they are constructed in the form of riboswitches to achieve an interference with the intracellular environment through interactions with signal molecules. Based on its excellent signal sensing capability, the CRISPR signal sensor has the potential to be used to detect toxic substances in the environment. In addition, the most use of the CRISPR signal sensor is still concentrated in the medical field. The CRISPR sensors that sense the changes of proteins and RNA sequences at the genomic level shown in Figure 4 have various medical applications, including disease diagnosis and treatment. For example, when the cells are subjected to pathological conditions such as infection by pathogens, the RNA transcripts and protein markers exhibit a change in the amounts of expressions. Disease development or cell differentiation will also exhibit such specific changes at different stages. Therefore, the CRISPR signal sensors we designed for proteins and RNA transcripts can detect specific stages of diseases and may cure diseases by repairing cell signaling regulatory networks.

There are also some challenges for developing CRISPR signal sensors. Although sgRNA sensing intracellular RNA molecules is relatively easy to design because it only requires the base pairing principle, we must rely on RNA aptamers when different protein molecules within the cell are to be sensed. The number of RNA aptamers currently available to us is very limited. This requires further large-scale screening of RNA aptamers that bind to different protein molecules using emerging approaches^47^ and establish a detailed database. This will greatly benefit the reconstruction of the network of intracellular protein signaling molecules in the future. Another obvious problem is that the DNA coding sequence of the CRISPR system is relatively long and it is difficult to carry it into tissues through a gene therapy vector such as AAV, thereby limiting the further application of the CRISPR signal sensor to the treatment of diseases including cancers. But with the discovery of smaller Cas proteins and the advent of some simplified versions of CRISPR^48^, ^49^, this problem is gradually being solved. At the same time, the potential off-target effects of the CRISPR system cannot be ignored. Although the constructed signal sensors could conditionally control the activity of the CRISPR system, we still cannot guarantee that Cas proteins guide by sgRNA will not affect the off-target sites. However, many studies have shown that off-target effects could be prevented. The possible means include the design of highly specific sgRNA with predictive analysis^50^, ^51^, the construction of truncated sgRNA with various modifications^52^, ^53^ and the use of Cas proteins with high specificity^54^.

## Figures and Tables

**Table 1:** Applications of different types of cellular signal sensors.

Sensor types	Target	Cell type	Function and possible disadvantage	Modularity	References (PMID)
**P53 sensor**	Cellular P53 protein	HCT116 , MEFs, HEK293, LS123, WiDr, IMR90, BT-549, COLO320DM and 5637 cells	detecting cells that express common p53 mutations	Low	29133879 29957992
**Tango-TEV actuation module**	HCV; HIV; huntingtin gene (HTT)	HEK293FT; HeLa-based TZM-bl and Jurkat cells	initiating programmed transcriptional response when detecting target specific proteins	Medium	29760420
**Optical genetic switch**	Blue or red light	Neuro 2a; Neurons; HEK293; HeLa; hMSC-TERT and MCF-7 cells	Light-inducible spatiotemporal control of gene activation	Medium	23877069 29967137 22963237
**Antisense RNAs**	Theophylline; Tetracycline; β-catenin; VEGF;OPN and NF-kB	T24, 5637, and HEK-293 cells	regulating the expression of target transcripts in response to different cellular effectors; limited by the weak binding ability of antisense RNAs to mRNAs	High	29319503 15723047
**CRISPR-** **dCas9**	Theophylline; Tetracycline; Guanine; β-catenin; Ets-1; NF-kB and P53	T24,5637,HepG2 and HEK-293 cells	Sensing small molecules and cellular proteins; limited by the potential off-target effects	High	27595406 28656978 …
**CRISPR-** **dCas12a**	Theophylline; Tetracycline;	HEK-293 cells	Sensing small molecules and cellular proteins; limited by the available aptamers and receptors	High	29235474

## References

[1] Gordley RM, Bugaj LJ, Lim WA (2016). Modular engineering of cellular signaling proteins and networks. Current opinion in structural biology.

[2] Toda S, Blauch LR, Tang SKY, Morsut L, Lim WA (2018). Programming self-organizing multicellular structures with synthetic cell-cell signaling. Science (New York, NY).

[3] Ye H, Xie M, Xue S, Charpin-El Hamri G, Yin J, Zulewski H (2017). Self-adjusting synthetic gene circuit for correcting insulin resistance. Nature biomedical engineering.

[4] Guy CP, Majernik AI, Chong JP, Bolt EL (2004). A novel nuclease-ATPase (Nar71) from archaea is part of a proposed thermophilic DNA repair system. Nucleic acids research.

[5] Makarova KS, Aravind L, Grishin NV, Rogozin IB, Koonin EV (2002). A DNA repair system specific for thermophilic Archaea and bacteria predicted by genomic context analysis. Nucleic acids research.

[6] Mojica FJ, Diez-Villasenor C, Soria E, Juez G (2000). Biological significance of a family of regularly spaced repeats in the genomes of Archaea, Bacteria and mitochondria. Molecular microbiology.

[7] Jinek M, Chylinski K, Fonfara I, Hauer M, Doudna JA, Charpentier E (2012). A programmable dual-RNA-guided DNA endonuclease in adaptive bacterial immunity. Science (New York, NY).

[8] Kocak DD, Josephs EA, Bhandarkar V, Adkar SS, Kwon JB, Gersbach CA (2019). Increasing the specificity of CRISPR systems with engineered RNA secondary structures.

[9] Qi LS, Larson MH, Gilbert LA, Doudna JA, Weissman JS, Arkin AP (2013). Repurposing CRISPR as an RNA-guided platform for sequence-specific control of gene expression. Cell.

[10] Zetsche B, Volz SE, Zhang F (2015). A split-Cas9 architecture for inducible genome editing and transcription modulation. Nature biotechnology.

[11] Cebrian-Serrano A, Davies B (2017). CRISPR-Cas orthologues and variants: optimizing the repertoire, specificity and delivery of genome engineering tools. Mammalian genome: official journal of the International Mammalian Genome Society.

[12] Gao L, Cox DBT, Yan WX, Manteiga JC, Schneider MW, Yamano T (2017). Engineered Cpf1 variants with altered PAM specificities. Nature biotechnology.

[13] Zetsche B, Gootenberg JS, Abudayyeh OO, Slaymaker IM, Makarova KS, Essletzbichler P (2015). Cpf1 is a single RNA-guided endonuclease of a class 2 CRISPR-Cas system. Cell.

[14] Liu Y, Han J, Chen Z, Wu H, Dong H, Nie G (2017). Engineering cell signaling using tunable CRISPR-Cpf1-based transcription factors. Nature communications.

[15] Gilbert LA, Larson MH, Morsut L, Liu Z, Brar GA, Torres SE (2013). CRISPR-mediated modular RNA-guided regulation of transcription in eukaryotes. Cell.

[16] Zhang X, Wang J, Cheng Q, Zheng X, Zhao G, Wang J (2017). Multiplex gene regulation by CRISPR-ddCpf1. Cell discovery.

[17] Bikard D, Jiang W, Samai P, Hochschild A, Zhang F, Marraffini LA (2013). Programmable repression and activation of bacterial gene expression using an engineered CRISPR-Cas system. Nucleic acids research.

[18] Hilton IB, D'Ippolito AM, Vockley CM, Thakore PI, Crawford GE, Reddy TE (2015). Epigenome editing by a CRISPR-Cas9-based acetyltransferase activates genes from promoters and enhancers. Nature biotechnology.

[19] Liu X, Zhang Y, Chen Y, Li M, Zhou F, Li K (2017). In Situ Capture of Chromatin Interactions by Biotinylated dCas9. Cell.

[20] Chen B, Hu J, Almeida R, Liu H, Balakrishnan S, Covill-Cooke C (2016). Expanding the CRISPR imaging toolset with Staphylococcus aureus Cas9 for simultaneous imaging of multiple genomic loci. Nucleic acids research.

[21] Li Y, Li S, Wang J, Liu G (2019). CRISPR/Cas Systems towards Next-Generation Biosensing. Trends in biotechnology.

[22] Hu JH, Miller SM, Geurts MH, Tang W, Chen L, Sun N (2018). Evolved Cas9 variants with broad PAM compatibility and high DNA specificity. Nature.

[23] Kleinstiver BP, Prew MS, Tsai SQ, Nguyen NT, Topkar VV, Zheng Z (2015). Broadening the targeting range of Staphylococcus aureus CRISPR-Cas9 by modifying PAM recognition. Nature biotechnology.

[24] Mircetic J, Dietrich A, Paszkowski-Rogacz M, Krause M, Buchholz F (2017). Development of a genetic sensor that eliminates p53 deficient cells. Nature communications.

[25] Zhan H, Xie H, Zhou Q, Liu Y, Huang W (2018). Synthesizing a Genetic Sensor Based on CRISPR-Cas9 for Specifically Killing p53-Deficient Cancer Cells. ACS synthetic biology.

[26] Siciliano V, DiAndreth B, Monel B, Beal J, Huh J, Clayton KL (2018). Engineering modular intracellular protein sensor-actuator devices. Nature communications.

[27] Konermann S, Brigham MD, Trevino A, Hsu PD, Heidenreich M, Cong L (2013). Optical control of mammalian endogenous transcription and epigenetic states. Nature.

[28] Shao J, Wang M, Yu G, Zhu S, Yu Y, Heng BC (2018). Synthetic far-red light-mediated CRISPR-dCas9 device for inducing functional neuronal differentiation. Proceedings of the National Academy of Sciences of the United States of America.

[29] Polstein LR, Gersbach CA (2012). Light-inducible spatiotemporal control of gene activation by customizable zinc finger transcription factors. Journal of the American Chemical Society.

[30] Liu Y, Li J, Chen Z, Huang W, Cai Z (2018). Synthesizing artificial devices that redirect cellular information at will.

[31] Tapsin S, Sun M, Shen Y, Zhang H, Lim XN, Susanto TT (2018). Genome-wide identification of natural RNA aptamers in prokaryotes and eukaryotes. Nature communications.

[32] Mandal M, Breaker RR (2004). Adenine riboswitches and gene activation by disruption of a transcription terminator. Nature structural & molecular biology.

[33] Batey RT, Gilbert SD, Montange RK (2004). Structure of a natural guanine-responsive riboswitch complexed with the metabolite hypoxanthine. Nature.

[34] Vincent HA, Robinson CJ, Wu MC, Dixon N, Micklefield J (2014). Generation of orthogonally selective bacterial riboswitches by targeted mutagenesis and in vivo screening. Methods in molecular biology (Clifton, NJ).

[35] Gilbert SD, Reyes FE, Edwards AL, Batey RT (2009). Adaptive ligand binding by the purine riboswitch in the recognition of guanine and adenine analogs. Structure (London, England: 1993).

[36] McCown PJ, Liang JJ, Weinberg Z, Breaker RR (2014). Structural, functional, and taxonomic diversity of three preQ1 riboswitch classes. Chemistry & biology.

[37] Wu MC, Lowe PT, Robinson CJ, Vincent HA, Dixon N, Leigh J (2015). Rational Re-engineering of a Transcriptional Silencing PreQ1 Riboswitch. Journal of the American Chemical Society.

[38] Lee ER, Blount KF, Breaker RR (2009). Roseoflavin is a natural antibacterial compound that binds to FMN riboswitches and regulates gene expression. RNA biology.

[39] Liu Y, Zhan Y, Chen Z, He A, Li J, Wu H (2016). Directing cellular information flow via CRISPR signal conductors. Nature methods.

[40] Galizi R, Jaramillo A (2019). Engineering CRISPR guide RNA riboswitches for in vivo applications. Current opinion in biotechnology.

[41] Kundert K, Lucas JE, Watters KE, Fellmann C, Ng AH, Heineike BM (2019). Controlling CRISPR-Cas9 with ligand-activated and ligand-deactivated sgRNAs. Nature communications.

[42] Siu KH, Chen W (2019). Riboregulated toehold-gated gRNA for programmable CRISPR-Cas9 function. Nature chemical biology.

[43] Ferry QR, Lyutova R, Fulga TA (2017). Rational design of inducible CRISPR guide RNAs for de novo assembly of transcriptional programs. Nature communications.

[44] Tang W, Hu JH, Liu DR (2017). Aptazyme-embedded guide RNAs enable ligand-responsive genome editing and transcriptional activation. Nature communications.

[45] Liu Y, Kannegulla A, Wu B, Cheng LJ (2018). Quantum Dot Fullerene-Based Molecular Beacon Nanosensors for Rapid, Highly Sensitive Nucleic Acid Detection. ACS applied materials & interfaces.

[46] Saragliadis A, Hartig JS (2013). Ribozyme-based transfer RNA switches for post-transcriptional control of amino acid identity in protein synthesis. Journal of the American Chemical Society.

[47] Boussebayle A, Torka D, Ollivaud S, Braun J, Bofill-Bosch C, Dombrowski M (2019). Next-level riboswitch development-implementation of Capture-SELEX facilitates identification of a new synthetic riboswitch. Nucleic acids research.

[48] Zhan H, Zhou Q, Gao Q, Li J, Huang W, Liu Y (2019). Multiplexed promoterless gene expression with CRISPReader. Genome biology.

[49] Senis E, Fatouros C, Grosse S, Wiedtke E, Niopek D, Mueller AK (2014). CRISPR/Cas9-mediated genome engineering: an adeno-associated viral (AAV) vector toolbox. Biotechnology journal.

[50] Tsai SQ, Zheng Z, Nguyen NT, Liebers M, Topkar VV, Thapar V (2015). GUIDE-seq enables genome-wide profiling of off-target cleavage by CRISPR-Cas nucleases. Nature biotechnology.

[51] Doench JG, Fusi N, Sullender M, Hegde M, Vaimberg EW, Donovan KF (2016). Optimized sgRNA design to maximize activity and minimize off-target effects of CRISPR-Cas9. Nature biotechnology.

[52] Kocak DD, Josephs EA, Bhandarkar V, Adkar SS, Kwon JB, Gersbach CA (2019). Increasing the specificity of CRISPR systems with engineered RNA secondary structures. Nature biotechnology.

[53] Fu Y, Sander JD, Reyon D, Cascio VM, Joung JK (2014). Improving CRISPR-Cas nuclease specificity using truncated guide RNAs. Nature biotechnology.

[54] Pickar-Oliver A, Gersbach CA (2019). The next generation of CRISPR-Cas technologies and applications. Nature reviews Molecular cell biology.

